# Medication equivalent dose program in R

**DOI:** 10.1111/pcn.13817

**Published:** 2025-03-31

**Authors:** Shinsuke Koike, Yoji Hirano, Shinichiro Nakajima, Kentaro Morita

**Affiliations:** ^1^ University of Tokyo Institute for Diversity and Adaptation of Human Mind The University of Tokyo Tokyo Japan; ^2^ Center for Evolutionary Cognitive Sciences, Graduate School of Arts and Sciences The University of Tokyo Tokyo Japan; ^3^ The International Research Center for Neurointelligence (WPI‐IRCN) The University of Tokyo Institutes for Advanced Study (UTIAS) Tokyo Japan; ^4^ Department of Psychiatry, Division of Clinical Neuroscience, Faculty of Medicine University of Miyazaki Miyazaki Japan; ^5^ Department of Neuropsychiatry Keio University, School of Medicine Tokyo Japan; ^6^ Department of Rehabilitation University of Tokyo Hospital Tokyo Japan

Therapeutic drugs for psychiatric disorders are designed to target specific brain functions, resulting in alterations not only in psychiatric symptoms but also in brain structure and function, cognitive and psychomotor function, and other domains. Consequently, research on psychiatric disorders should consider the prescription and utilization of psychiatric drugs by participants at the time of their assessment. Antipsychotics, antidepressants, anxiolytics and hypnotics, and anticholinergic agents are currently quantified in equivalent doses to chlorpromazine, imipramine, diazepam, and biperiden, respectively.^1^ L‐dopa equivalent dose is used to estimate the quantity of dopamine agonists for the treatment of Parkinson's disease in neurology.^2^

Despite the development of various tools for calculating equivalent doses,^3^ their flexible application has been hindered by variability in approved drugs and trade names across countries and regions. To facilitate the work of researchers and research administrators while minimizing errors, it would be advantageous to develop a user‐friendly list of equivalent doses tailored to each research facility, accommodate diverse input formats for existing prescription data, and provide suggestions for newly acquired information.

We developed a program and input support tool capable of calculating various medication‐equivalent doses (https://github.com/koikelab/medication_eq_R). This program can process an xlsx‐formatted file containing identification numbers, survey dates, and drug name‐dose pairs. Although it prepares four equivalent doses as an exemplar in Japanese medical contexts (Table 1),^1^, ^4^ it can subsequently calculate other equivalent doses^5^, ^6^ by editing the template. For example, amisulpride is not listed in the default file because this drug is not approved in Japan, but users can add amisulpride to the list according to previous literature.^5^ Actually, it's the equivalent dose of vortioxetine has not been determined, and we added the equivalent dose according to a systematic review.^7^

**Table 1:** Example of equivalent doses originally implemented in the program

Name	CP	CP old	IMP	DZP	BPD
Aripiprazole	4.0				
Asenapine	2.5				
Chlorpromazine	100.0	100.0			
Risperidone	1.0				
Zotepine	66.0	66.0			
Haloperidol injection	1.00	1.00			
Olanzapine injection	2.25				
Haloperidol decanoate (4w)	30.00	30.00			
Aripiprazole once monthly (4w)	100.00				
Amantadine					100.00
Biperiden					2.00
Trihexyphenidyl					4.00
Amitriptyline			150		
Imipramine			150		
Paroxetine			40		
Sertraline			100		
Venlafaxine			150		
Alprazolam				0.80	
Diazepam				5.00	
Etizolam				1.50	
Zopiclone				7.50	
Vortioxetine^7^			20		

This table was extracted from the source file in the program, which was originally made from previous studies.^1^, ^4^ CP old indicates chlorpromazine equivalent doses for first‐generation antipsychotics.

Abbreviations: BPD, biperiden; CP, chlorpromazine; DZP, diazepam; IMP, imipramine.

The output xlsx‐formatted files include equivalent doses for each drug name and dose as well as the total equivalent doses for each identification number and survey date. Drug name‐dose pairs in the input file can be arranged either vertically or horizontally. Moreover, by referring to the drug list of the National Health Insurance,^8^ the input drug names are converted to standard English drug names, irrespective of whether they are trade names, drug names, or common names in the local language. By modifying the source file containing this information, it is possible to efficiently calculate the equivalent doses and convert them into standard English drug names. Furthermore, by revising the list of standard name and equivalent dose pairs, the program can calculate the revised and new equivalent doses.

The input support tool utilizes the standard suggestion function implemented in Microsoft Excel to facilitate the input of drug names in the source file through recommendations. This methodology enhances input efficiency and mitigates errors. The tool can search a database of Japanese product names and drug names in half‐width Roman characters, as well as standard English drug names, to expedite the input process.

The goal of this research program is to compile comprehensive medication data and utilize this information to investigate the biological, psychological, and social implications of pharmaceutical interventions. Through the collection of detailed information regarding individual pharmacological agents, it may be feasible to promote the research of drug effect in the future.

## Disclosure statement

Shinsuke Koike is an Editorial Board member of *Psychiatry and Clinical Neurosciences* and a co‐author of this article. Yoji Hirano is an Art Advisor of *Psychiatry and Clinical Neurosciences* and a co‐author of this article. To minimize bias, they were excluded from all editorial decision‐making related to the acceptance of this article for publication.

## Author contributions

S.K., Y.H., and S.N. contributed to the design of the work. S.K. contributed to writing the program and a draft manuscript. All authors contributed to revising the program and manuscript critically, and gave final approval of the submitted version.
